# Supplementary material for: Learning curve and surgical outcome of robotic assisted colorectal surgery with ERAS program
Source: Sci Rep. 2022 Nov 29;12:20566. doi: 10.1038/s41598-022-24665-w (PMC9709162; doi:10.1038/s41598-022-24665-w)
Supplement: Supplementary file 1 — Supplementary Information 1. [file 41598_2022_24665_MOESM1_ESM.zip › LSC Raw data-Ñ[▒K/066/P_ñw╝╨░O▒Kñσ.pdf]

臺中榮民總醫院
臺中市臺灣大道四段1650號
病理部活體組織[illegible]
臨床醫學實驗及病理診斷中心 檢驗(查)報告

[redacted]

Pathologic diagnosis:
1. Decending colon, lapa left hemicolectomy --- Adenocarcinoma, moderately differentiated. Tumor invaded to mesocolic soft tissue, and metastasized to mesocolic lymph nodes (2/19). The bilateral cut ends are free of tumor.
2. Colon, cut end, distal, resection --- Unremarkable.
3. IMA root, dissection --- Unremarkable fibrovascular tissue.
4. Ovary and fallopian tube, left, salpingo-oophorectomy --- Unremarkable fallopian tube and ovary.

已電子簽章

敬告：本單僅供醫師參考使用，不具任何申請效力！！
請病患妥為保管，若不慎遺失需自負個人資料外洩責任！

Ancillary study for diagnosis:
IHC stains for MLH1, MSH2, PMS2 and MSH6 are done on section C. All above markers are preserved.

Prognostic and predictive factor:
1. Tumor size: 2.2x1.7 cm.
2. Depth of invasion: Pericolorectal tissue.
3. Histologic grade: G2: Moderately differentiated.
4. Angiolymphatic invasion: Present.
5. Perineural invasion: Not identified.
6. Tumor deposits (discontinuous extramural extension): Present in mesocolic soft tissue, specify number of deposits: 2.
7. Macroscopic tumor perforation: Not identified.
8. Surgical Margins: a) Proximal Margin: Uninvolved by invasive carcinoma, distance of tumor from margin: 16.3 cm. b) Distal Margin: Uninvolved by invasive carcinoma, distance of tumor from margin: 10 cm. c) Radial or Mesenteric Margin: Uninvolved by invasive carcinoma, distance of tumor from margin: 5 mm.
9. Lymph node status: Uninvolved (2/19).
10. Extranodal involvement: Not identified.
11. Tumor regression grading S/P CCRT: No known presurgical therapy.
12. Type of polyp in which invasive carcinoma arose: Not identified.
13. Additional pathologic findings: Not identified.
14. Pathological TNM Stage: pT3N1a(According to the eighth edition, American Joint Committee on Cancer Staging Guidelines for Tumors).
15. TNM descriptors: Not applicable.

Gross description:
The specimen consists of 1) a segment of colon, 28.7 cm in length and 5.6 cm in proximal cut end circumference, 6.8 cm in distal circumference, and mesocolic soft tissue. A gray tan firm annular elevated and centrally ulcerative tumor, 2.2x1.7 cm, is noted, 10 cm to the distal cut end. On cross section, the tumor invades through the mesocolic soft tissue to serosal surface, and 2 cm to the radial margin. The both cut ends are free of tumor grossly. 2) IMA root, a piece of tan yellow soft tissue, 2.3x2x0.6 cm. 3) distal cut end, a piece of tan mucosa-covered soft tissue, 3x2x0.8 cm. 4) a part of fallopian tube, 3x1.1 cm, and an ovary, 2.3x1x0.8 cm, are unremarkable grossly. Representative part for section: A) proximal cut end B) distal cut end C-D) tumor E) radial margin (inked yellow) F-G) specimen 2-3 H-J) specimen 4 (H: fallopian tube, J: ovary) K-Z) lymph nodes.

Note:
1. The interpretation of MMR panel and recommended studies are as below: (1) No loss of nuclear expression of MMR proteins: low probability of microsatellite instability-high (MSI-H). (2) Please note: There are exceptions to the above IHC interpretations. These results should not be considered in isolation, and clinical correlation with genetic counseling is recommended to assess the need for germline testing.
2. This case has been peer reviewed by two doctors.

Erratum: 2020.03.03

Prognostic and predictive factor:
9. Lymph node status: Ininvolved (2/19).
14. Pathological TNM Stage: pT3N1b(According to the eighth edition, American Joint Committee on Cancer Staging Guidelines for Tumors).
#T-59300_2 #M-81403_2 2 1105 S0ER0F
#T-C4560_2 #M-81406_2 9 1105 000000
